# Percutaneous Transhepatic Embolization of Ectopic Varices in a Patient With Portal Hypertension Presenting With Hemorrhagic Shock

**DOI:** 10.7759/cureus.18209

**Published:** 2021-09-23

**Authors:** Zachary Ohs, Matthew Jones, Neil Sharma, Kristian Loveridge

**Affiliations:** 1 Interventional Radiology, Detroit Medical Center, Detroit, USA

**Keywords:** ectopic varices management, mesenteric varices, bleeding varices, hemoperitoneum, decompressive shunt, colonic varices, portal hypertension, ectopic varices

## Abstract

Varices secondary to portal hypertension in the setting of liver cirrhosis typically occur in the gastroesophageal region. Management guidelines for bleeding gastroesophageal varices are well established in the literature. Ectopic varices that occur outside of this typical location are an uncommon complication of portal hypertension. Rarely, these varices can result in life-threatening hemorrhage. Management guidelines of ectopic variceal bleeds are not yet standardized as cases are rare and treatment approach in the literature has historically varied. We present an interesting case of a 37-year-old patient with alcoholic liver disease and cirrhosis who developed spontaneous hemorrhage and shock from bleeding ectopic varices. This report exemplifies how coil embolization via a percutaneous transhepatic approach can be used to manage ectopic variceal bleeds in the setting of hemorrhagic shock.

## Introduction

Porto-systemic decompressive varices in the setting of portal hypertension secondary to liver cirrhosis are a well-documented phenomenon with the most common locations of shunts being esophageal and gastric [1]. As portal hypertension worsens, patients are at a greater risk of the feared complication of bleeding varices. Variceal hemorrhage carries with it high mortality and morbidity rates and thus it is important to understand its pathophysiology and management [2]. Ectopic varices, those that occur outside of these common regions, make up only 1-5% of bleeding varices in patients with portal hypertension. Their management is thus less well established with no clear guidelines [3]. Localizing bleeding ectopic varices is often clinically difficult and management is largely based upon case series and reports of individual patients [1].

We present a case of a 37-year-old patient with hemorrhagic shock from bleeding right colic varices in the setting of liver cirrhosis secondary to alcohol abuse. This case provides an example of endovascular treatment of bleeding ectopic varices using interventional techniques, which may serve as a guide for future patients with a similar presentation.

## Case presentation

A 37-year-old female with a past medical history significant for alcohol use disorder and hyperlipidemia presented to the emergency department with one day of weakness, abdominal pain, and distension.

Vitals on admission are illustrated in Table 1.

**Table 1:** Vital signs on admission

Blood pressure	78/48 (low)
Heart rate	101 (high)
Oxygen saturation	98% on room air
Respiratory rate	16
Temperature	37.0

**Figure 1:**
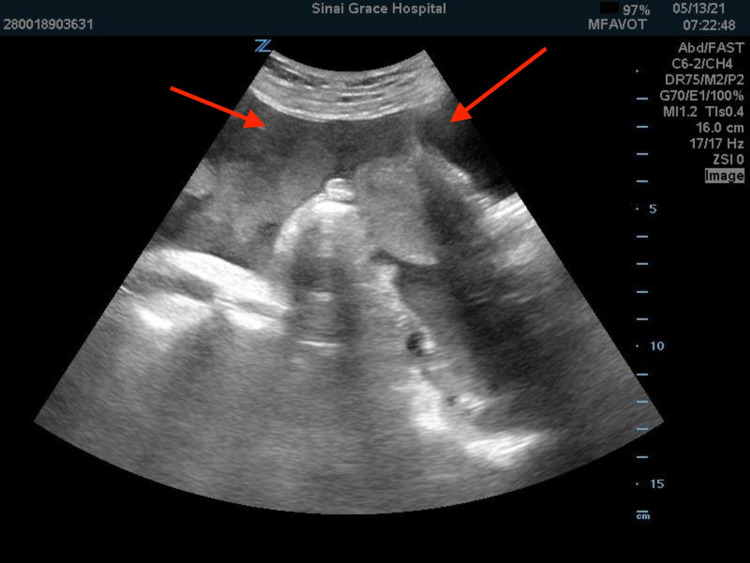
Bedside FAST exam demonstrated complex echogenic fluid in the abdomen, concerning for hemoperitoneum (arrows). FAST, focused assessment with sonography in trauma.

Physical examination was positive for scleral icterus and conjunctival pallor as well as diffuse abdominal tenderness to deep and superficial palpation with guarding, but no rebound tenderness. Bedside ultrasound was performed showing hyperdynamic cardiac function, 100% collapsible inferior vena cava, and complex free fluid in the abdomen (Figure 1).

**Figure 2:**
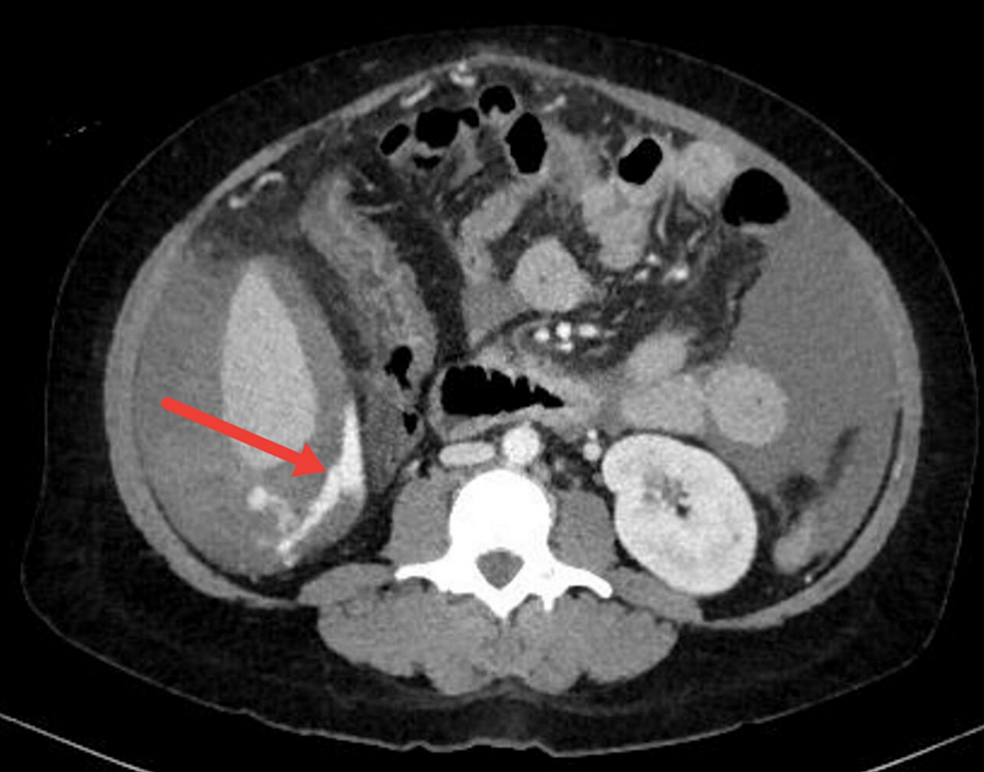
Axial CT image demonstrates an active extravasation of blood inferior to the right hepatic lobe (arrow). CT, computed tomography.

**Figure 3:**
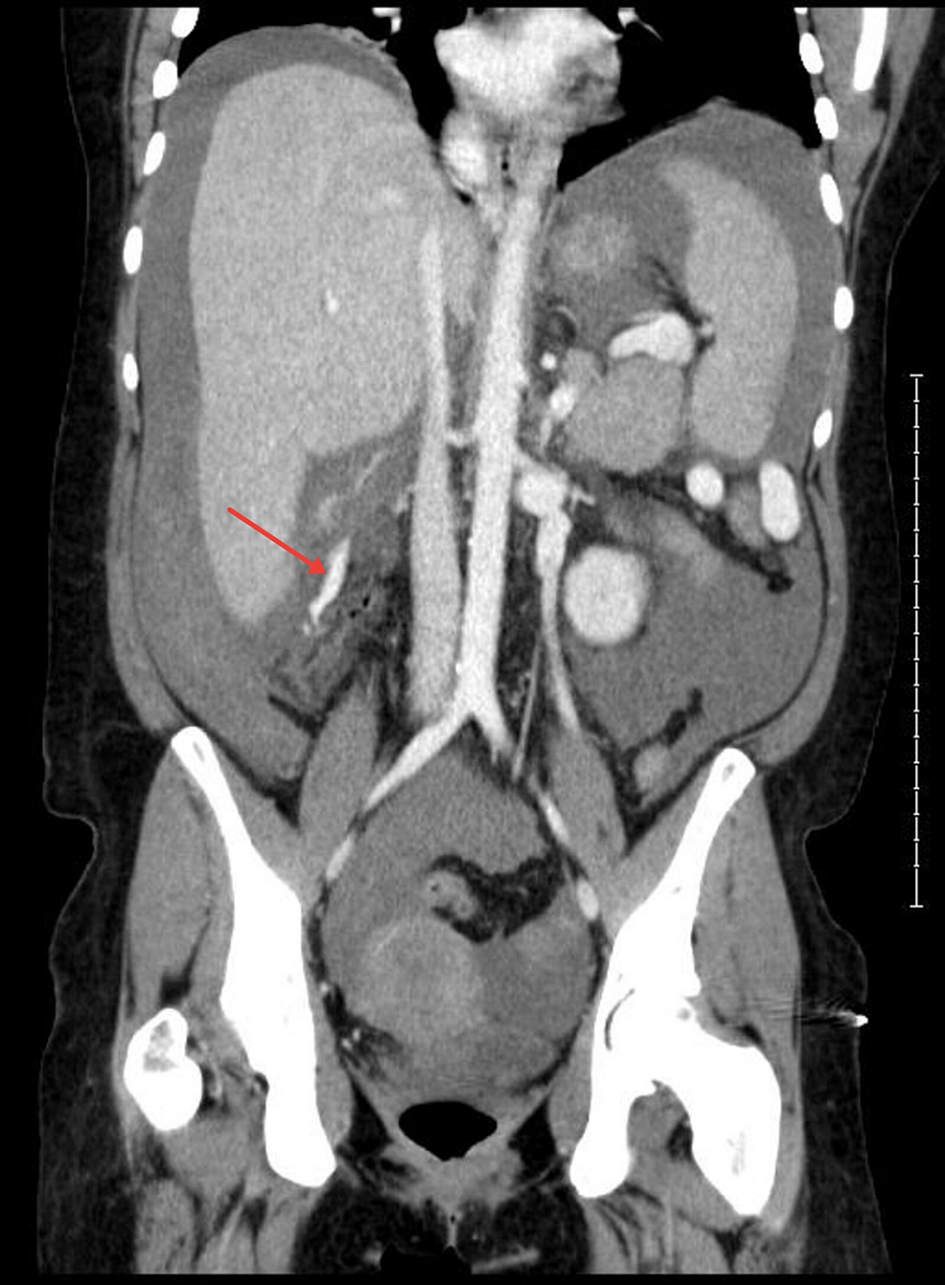
Coronal CT image shows an active extravasation of blood inferior to the right hepatic lobe (arrow). CT, computed tomography.

At this point, the differential diagnosis was broad but included hemorrhagic shock and sepsis. Labs were drawn, and are illustrated in Table 2. A computed tomography (CT) scan of the abdomen demonstrated cirrhotic liver with evidence of portal hypertension and active bleeding from right colic varices with hemoperitoneum (Figures 2, 3). Interventional Radiology was consulted for potential embolization.

**Table 2:** Pertinent labs

Total bilirubin	10.2 (high)
Alanine aminotransferase	11
Aspartate aminotransferase	77 (high)
Alkaline phosphatase	63
Total protein	6.6
Albumin	2.9 (low)
Hemoglobin	7.2 (low)
Platelets	93 (low)
Lactate	4.6 (high)
Hemoglobin repeat	5.2 (low)

**Figure 4:**
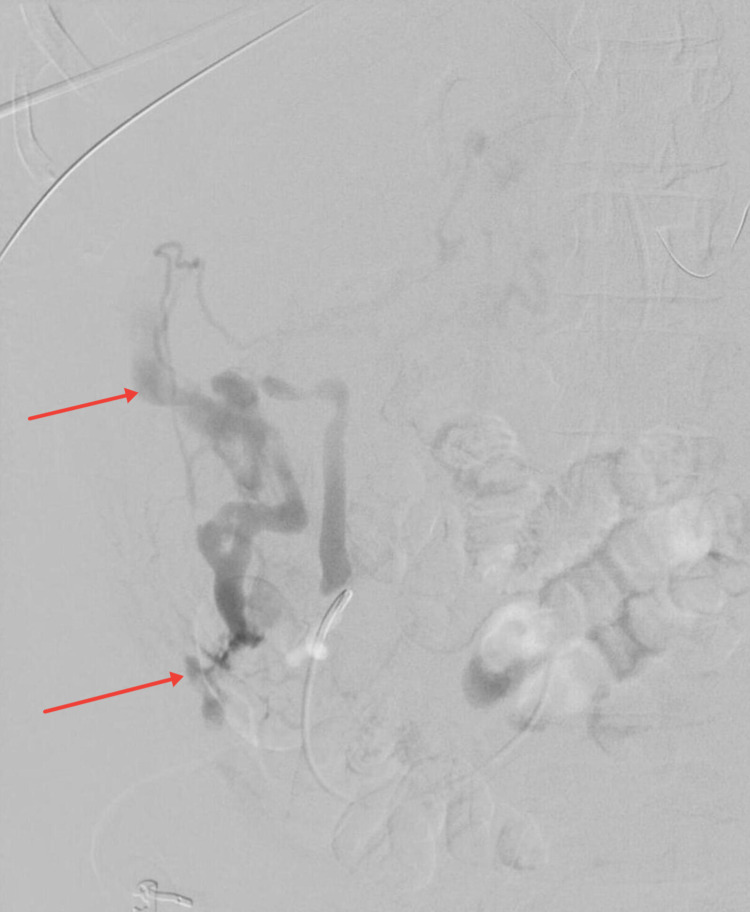
Angiogram demonstrates active extravasation from intraperitoneal varices and pooling of contrast in the right upper quadrant.

From a percutaneous transhepatic approach, a right portal vein branch was directly accessed. Portal venography showed hepatofugal flow through the main portal vein. Selective venography of the superior mesenteric vein was performed demonstrating retrograde flow through right colic and ileocolic veins. A mesenteric (right colic vein) to inferior vena cava decompressive shunt was identified, with focal extravasation and pooling of contrast in the right upper quadrant adjacent to the inferior right hepatic lobe (Figure 4).

**Figure 5:**
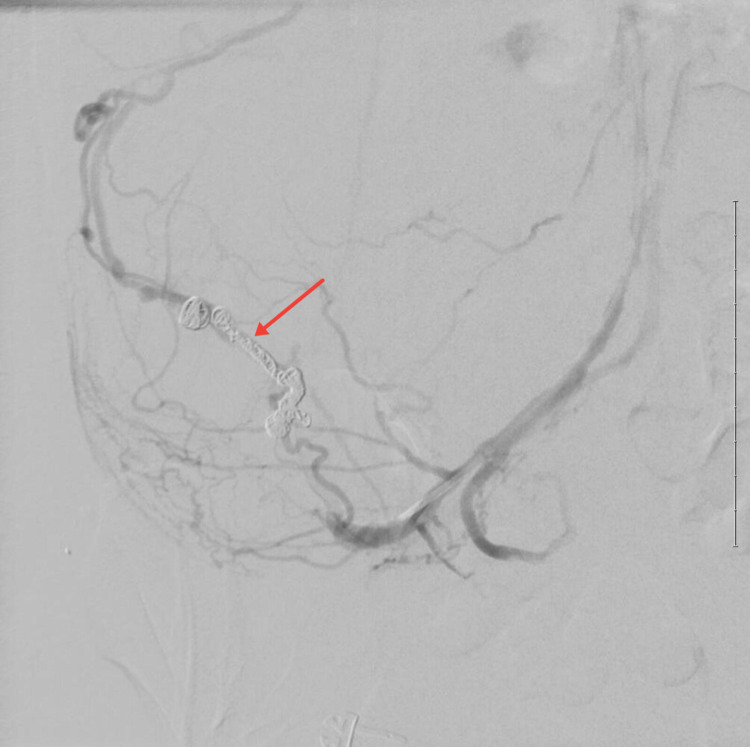
Repeat angiogram after coiling demonstrates no active extravasation.

Using a microcatheter, access across the point of extravasation was achieved. Coils were deployed from distal to proximal across the site of bleeding, employing the “front door, back door” technique. Embolization was augmented with Gelfoam to promote stasis of flow within the varix. Follow-up angiography demonstrated occlusion of the varix with no further extravasation (Figure 5).

The anesthesia team reported decreased tidal volumes, and a tense abdomen was observed. A paracentesis was performed, yielding 3 liters of sanguineous ascites resulting in subsequent improvement in tidal volume.

The patient was admitted to the intensive care unit for ongoing hemodynamic monitoring. Given the continued presence of portal hypertension, it was felt that she may benefit from future intervention such as partial splenic artery embolization or transjugular intrahepatic portosystemic shunt (TIPS) creation.

Following the procedure, the patient’s hemoglobin remained stable. However, the patient expired several days after the embolization due to sepsis and multiorgan dysfunction.

## Discussion

Ectopic varices are dilated porto-systemic decompressive veins found outside of the cardio-esophageal junction and are rare but serious complications of portal hypertension. Intraperitoneal varices, a subgroup of ectopic varices, are exceedingly rare. They most often arise as a result of portal hypertension, commonly secondary to alcohol-induced liver cirrhosis [4]. While this was the case in the patient discussed above, there are other less common causes of portal hypertension leading to intraperitoneal varices. Thrombosis is the main pre-hepatic cause and is due to conditions such as hypercoagulable states and thrombophilic disorders. The literature most often references myeloproliferative disorders, antiphospholipid syndrome, deficiency of protein C, S, and antithrombin III, and mutation of factor V Leyden as common causes of thrombosis [4]. The patient described in this case suffered from the most common cause of intrahepatic portal hypertension, alcohol induced, but other causes do include viral, metabolic, and toxic.

Since cases of intraperitoneal varices secondary to portal hypertension are rare, the data behind management for hemorrhagic varices in this setting are even more limited. No specific guidelines elucidate ideal management, likely in part due to the fact that ectopic venous shunting pathways are so varied. Liu et al. described a case study with successful embolization of ectopic varices that drained into the right renal vein with balloon-occluded technique, which indeed is an unusual scenario [5]. Another study examined the efficacy of TIPS in ectopic variceal bleeding via a multicenter retrospective examination, and while they only had one patient with a cecal bleed, the patient did experience a positive outcome of no future bleeding [6]. Another case report described successful stabilization in a patient with hemorrhagic ectopic varices with coil embolization after TIPS procedure [7]. It is important to note that percutaneous embolization carries with it risks that need to be weighed with the benefits in each individual case. Complications include transient fever, nausea, and pain of post-embolization syndrome or less commonly vessel injury, infection, or thromboembolism. It is critical to determine if the patient is on anticoagulants/antiplatelet medications as, depending on the medication and procedure, these may need to be stopped prior to the treatment [8]. 

In our case, the patient underwent percutaneous transhepatic coil embolization alone, with a successful technical outcome. Indication to perform embolization was strictly to achieve hemostasis and stabilize the patient. TIPS was not performed at this time, in part due to the patient’s high score as calculated by using the Model For End-Stage Liver Disease. Although the patient expired during her hospitalization, she did not have signs of further bleeding. This demonstrates that initial management of bleeding ectopic varices may be achieved with interventional techniques until the underlying portal hypertension can be addressed [9].

## Conclusions

Ectopic varices are an uncommon sequela of portal hypertension, and hemorrhage of these varices can be catastrophic. With regard to this specific clinical entity, best practices have yet to be well established. However, given this case and other similar cases reported in the literature, we propose that interventional management may be a safe and effective first step in the treatment of patients with portal hypertension and bleeding ectopic varices.
